# Fabrication and Electrical Characterization of Low-Temperature Polysilicon Films for Sensor Applications

**DOI:** 10.3390/mi16010057

**Published:** 2024-12-31

**Authors:** Filipa C. Mota, Inês S. Garcia, Aritz Retolaza, Dimitri E. Santos, Patrícia C. Sousa, Diogo E. Aguiam, Rosana A. Dias, Carlos Calaza, Alexandre F. Silva, Filipe S. Alves

**Affiliations:** 1 International Iberian Nanotechnology Laboratory, 4715-330 Braga, Portugal; ines.garcia@inl.int (I.S.G.); aritz.retolaza@inl.int (A.R.); dimitri.santos@inl.int (D.E.S.); patricia.sousa@inl.int (P.C.S.); diogo.aguiam@inl.int (D.E.A.); rosana.dias@inl.int (R.A.D.); carlos.calaza@inl.int (C.C.); 2 Department of Industrial Electronics, University of Minho, 4710-057 Braga, Portugal; asilva@dei.uminho.pt

**Keywords:** metal-induced crystallization, polysilicon, low-temperature, gauge factor, temperature coefficient of resistance, polyimide

## Abstract

The development of low-temperature piezoresistive materials provides compatibility with standard silicon-based MEMS fabrication processes. Additionally, it enables the use of such material in flexible substrates, thereby expanding the potential for various device applications. This work demonstrates, for the first time, the fabrication of a 200 nm polycrystalline silicon thin film through a metal-induced crystallization process mediated by an AlSiCu alloy at temperatures as low as 450 °C on top of silicon and polyimide (PI) substrates. The resulting polycrystalline film structure exhibits crystallites with a size of approximately 58 nm, forming polysilicon (poly-Si) grains with diameters between 1–3 µm for Si substrates and 3–7 µm for flexible PI substrates. The mechanical and electrical properties of the poly-Si were experimentally conducted using microfabricated test structures containing piezoresistors formed by poly-Si with different dimensions. The poly-Si material reveals a longitudinal gauge factor (GF) of 12.31 and a transversal GF of −4.90, evaluated using a four-point bending setup. Additionally, the material has a linear temperature coefficient of resistance (TCR) of −2471 ppm/°C. These results illustrate the potential of using this low-temperature film for pressure, force, or temperature sensors. The developed film also demonstrated sensitivity to light, indicating that the developed material can also be explored in photo-sensitive applications.

## 1. Introduction

Flexible micro-electro-mechanical systems (MEMS) technologies have advanced over the past years, exploiting the ability to conform sensor systems to non-planar surfaces [1] in different applications, ranging from health monitoring devices [2,3] to robotics [4,5]. Piezoresistive transduction has gained popularity among various sensing mechanisms due to its simple manufacturing processes, high sensitivity, and wide detection range [6,7]. This mechanism employs semiconductors and metals as sensing elements [8,9,10], with semiconductors exhibiting a significantly higher gauge factor (GF) than metals due to variations in the material’s resistivity [11]. For instance, monocrystalline silicon can achieve a GF of 157 [12] while polycrystalline silicon (poly-Si or polysilicon) can reach up to 77 [13]. Despite the lower but still significant sensitivity to strain, the piezoresistive properties of poly-Si can be fine-tuned by the variation of the doping level and its grain size. Additionally, this material has the ability to be deposited/grown on different substates, such as thin glass [14], polyimide [15] and SU-8 [16], as randomly oriented crystals do not require lattice matching [17]. These properties, when combined, make this an attractive material to be used as a sensing element for flexible pressure/temperature-sensing applications.

The microfabrication process of poly-Si resistors on flexible substrates is constrained by the thermal stability of polymeric layers, which can withstand temperatures up to 500 °C, such as polyimide (PI) [18]. Poly-Si can be obtained by a two-step process that includes the direct deposition of amorphous silicon (a-Si) followed by an annealing step. While annealing can be performed by the incidence of a laser beam in the a-Si [19], this technique may incur high costs and is highly dependent on the laser energy intensity [20].

Alternatively, the poly-Si film can be grown through metal-induced crystallization (MIC), which consists of adding an auxiliary metal layer before the a-Si deposition step [21,22,23]. Here, this metal layer serves as a mediator of the crystallization process, enabling a temperature reduction in the annealing process when compared with solid-phase crystallization (SPC), a process in which the a-Si is annealed without a catalyst layer, requiring temperatures of around 600 °C [24]. Aluminum (Al) is commonly used in the MIC technique [25,26,27] as it enables the crystallization process at temperatures ranging from 180 °C to 577 °C [28].

Prior studies have demonstrated the potential of MIC with pure aluminum in various applications. For instance, Quintana et al. [14] developed a poly-Si film on ultra-thin flexible glass and polyimide for temperature sensors at a temperature of 400 °C for 1 h, achieving a TCR of −2500 ppm/°C for films developed on top of polyimide and −2800 ppm/°C for films on ultra-thin glass. Another work, by Tiwari and Chandra [29], investigated the piezoresistive properties of poly-Si films developed by aluminum-induced crystallization (AIC) at a temperature of 550 °C for 6 h, reporting a transversal gauge factor of 5 and longitudinal gauge factor of 18, validating the applicability of this material in high-sensitivity strain-based sensors. Wu et al. [30] employed the same technology with an annealing step at 400 °C for 1 h to develop flexible biomedical devices, achieving a GF of 10.3 and a TCR of −2700 ppm/°C, highlighting the multi-sensing capabilities of poly-Si. Furthermore, Patil et al. [31] developed a pressure sensor for tactile applications using poly-Si piezoresistors that had been developed by MIC at 400 °C for 1.5 h, reporting an estimated gauge factor ranging from 6.6 to 11.7.

Beyond pure Al, metallic alloys such as Ag_x_Al_1−x_ and Al_x_Zn_1−x_ are promising alternatives given the tunability of the crystallization thermal budget, and consequently the poly-Si structure, by varying the compound ratios [32]. Similarly, Al_2_Cu was also studied as a crystallization mediator by Moiseenko et al. [33] and it was observed that, while the crystallization kinetics were similar to that of typical aluminum-induced crystallization, the use of the alloy requires higher annealing temperatures when compared with pure Al. While these works validate the feasibility of using metallic alloys in MIC process, investigation into the electromechanical characteristics of poly-Si films mediated by alloys remains limited, presenting an opportunity for further research.

In MEMS microfabrication processes aluminum–silicon–copper alloy (AlSiCu) is frequently used to create electrical contacts in devices due to the material’s wide availability and high deposition rates [34,35]. As a result, using such an alloy as a crystallization mediator in the MIC process can shorten the fabrication time and ease the integration of this technique into existing fabrication processes. Additionally, as these technologies employ low-temperature processes (below 300 °C), they ensure that the piezoresistive properties of the resultant poly-Si film remain unchanged.

The use of AlSiCu as a crystallization mediator also influences the piezoresistive behavior of the resultant poly-Si film since both copper and aluminum act as p-type dopants. This type of dopant increases the positive charge carrier’s (holes) concentration in the material, decreasing its conductivity when compared with n-type dopants that induce a higher electron concentration due to their higher mobility. P-type piezoresistors are characterized by a positive longitudinal gauge factor, meaning that the resistance increases when the input signal has the same direction as the applied strain and a negative transversal gauge factor, where the resistance decreases when the input signal is applied to the resistor perpendicularly to the strain direction. Furthermore, the piezoresistive effect is often enhanced in p-type piezoresistors due to the additional band shape changes with the strain, an effect not observed for n-type piezoresistors [36].

This study presents the development of a poly-Si thin film on silicon and PI substrates using an AlSiCu alloy as a mediator based on the MIC mechanism. The main objective of this work is the study of a simple, low-temperature MEMS/flexible-substrate compatible crystallization method and the characterization of the structural, mechanical, and electrical properties of this poly-Si layer to be explored in potential sensing applications such as pressure and temperature sensors.

The experimental characterization of the film’s structure, namely the grain size and crystallographic orientation, was conducted using scanning electron microscopy (SEM) and X-ray diffraction (XRD) techniques. The electrical and mechanical properties of the developed poly-Si, namely the GF and the temperature coefficient of resistance (TCR), were extracted using dedicated microfabricated test structures containing poly-Si piezoresistors of different sizes. The poly-Si TCR was evaluated within a temperature range of 5 °C to 85 °C, while the gauge factor was measured over a strain range of 0 to 0.0008. Additionally, the photo-response of the material was evaluated by extracting the I–V characteristic under light and dark conditions for an input voltage ranging from −25 V to 25 V.

While primarily focused on the characterization of the structural and electromechanical properties of poly-Si films, this research validates the potential of AlSiCu as a crystallization mediator by achieving performance metrics, such as TCR and GF, comparable to those mediated by pure Al. These findings provide critical insights on the material’s behavior under various conditions such as temperature, strain and light, properties that are essential for the design and optimization of poly-Si based sensors as well as for the identification of potential errors that can impact their performance. Furthermore, this study demonstrates the compatibility of the MIC process mediated by AlSiCu with flexible substrates, emphasizing its potential for applications in wearable and flexible sensing systems.

## 2. Metal-Induced Crystallization Process

Solid-phase crystallization is a process where an a-Si layer deposited on a substrate is annealed at temperatures of around 500 °C to 700 °C for 24 to 48 h to ensure the complete crystallization of silicon [24]. In contrast, metal-induced crystallization is a technique in which the time and temperature required to achieve a crystalline Si film are reduced due to the presence of the metal layer that acts as a catalyst in the reaction.

**Figure 1:**
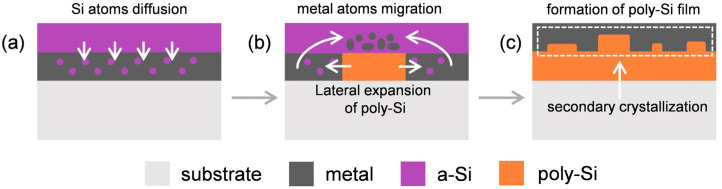
Illustration of the MIC process for non-silicide forming systems, which starts with (**a**) the diffusion of the Si atoms into the metal layer, followed by (**b**) the nucleation of the atoms into a crystalline structure, and finishing with (**c**) the achievement of a continuous poly-Si layer.

The MIC process involving aluminum (Al), silver (Ag), and gold (Au) results in the formation of a eutectic system where the metal acts as a catalyst, as depicted in Figure 1. According to Nast and Wenham [37], when the metal is in contact with a-Si, the Si bonds become unstable, resulting in a lower energy required for their rupture. Subsequently, silicon diffuses through the metallic layer, as shown in Figure 1a, until its solubility reaches its maximum value. At this stage, the diluted Si atoms nucleate with a polycrystalline structure and expand laterally, while the metal atoms migrate to the top, as shown in Figure 1b. Simultaneously, metal atoms on the top layer promote a secondary crystallization in which part of the a-Si atoms are used to form poly-Si islands. If the annealing conditions remain undisturbed, the crystallization process continues until all a-Si is consumed (Figure 1c). Aluminum is commonly employed in the crystallization process due to its reported effectiveness in a large temperature range (180 °C to 577 °C) and its role as a p+ dopant [26,38].

Metals like nickel (Ni), copper (Cu), and palladium (Pd) mediate the crystallization of silicon by forming a stable silicide. For instance, when using Ni, the Si atoms initially react with the Ni layer, forming a silicide compound (NiSi_2_). Because the chemical potential of Ni is lower than the chemical potential of Si at the NiSi_2_/a-Si interface, the formation of NiSi_2_ is favorable. However, the opposite is valid at the NiSi_2_/poly-Si interface, which leads to the formation of the poly-Si [39]. The same model can be used to describe the MIC process for the other types of compound-forming metals, such as Cu, that mediate the crystallization by the formation of Cu_3_Si. However, the use of Cu as MIC mediator requires a higher activation energy, approximately 2.2 eV [40], when compared with eutectic metals such as Al that require an activation energy of 1.4 eV [40] to induce the crystallization. This means that the thermal budget (temperature and time) required to obtain poly-Si is higher. The AlSiCu alloy used in this study is commercially available with a fixed composition of approximately 98% of Al, 1.5% of Si, and 0.5% of Cu. Due to the low concentration of Cu in the alloy composition, it is expected that the presence of this metal will have a minimal impact on the crystallization process. As for the Si content, it is anticipated to play a similar role to the a-Si atoms in promoting the crystallization step. Therefore, the thermodynamic behavior of this AlSiCu alloy can be approximated to that of pure Al.

Different growth parameters significantly impact the polycrystalline film structure (e.g., the system structure and annealing conditions). These parameters must be considered when aiming to develop a high-quality material, as follows:

- Layer thickness: The a-Si/metal ratio must always be greater than 1 to ensure a continuous crystalline film, as part of the a-Si is consumed during the secondary crystallization [37].
- Oxide layer: Following the deposition of Al, an oxide layer starts to form, acting as a diffusion barrier for Si atoms during annealing. This barrier slows the process and promotes lateral grain growth [41]. Minimizing the air exposure between the aluminum and a-Si depositions is crucial to prevent the formation of this layer and increase the speed of the crystallization process.
- Annealing conditions: Longer time steps and higher temperatures favor crystalline grain growth [42], while using inert gases in the annealing step accelerates the process. It is also stated that using H_2_ in the annealing process boosts the crystallization [43].

## 3. Materials and Methods

### 3.1. Development of Poly-Si

**Figure 2:**
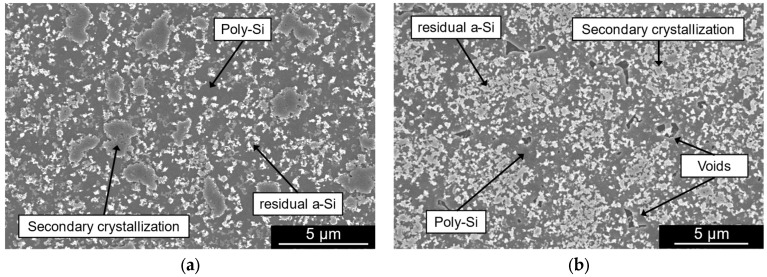
SEM pictures of the poly-Si film obtained by annealing the AlSiCu(200 nm)/a-Si(250 nm) at 450 °C for 8 h (**a**) on top of a Si wafer and (**b**) on top of PI 4110-HD layer.

This study focuses on the MIC method, which uses an AlSiCu alloy to achieve a poly-Si thin film on silicon and polyimide substrates. In this section, the processes of the tests towards the development of poly-Si on top of silicon and polymeric substrates are detailed. A 100 nm-thick SiO_2_ layer was deposited on top of a single-side polished (SSP) 8-in Si wafer by plasma-enhanced chemical vapor deposition (PECVD) with 1420 sccm of N_2_O and 392 sccm of N_2_ at 200 °C (using SPTS CVD) for electrical passivation. The thickness considered for this layer provides an effective electrical isolation between the substrate and the conductive poly-Si film and metallic pads while avoiding the thermal dissipation constraints that a thicker oxide layer would impose during the crystallization process due to its thermal insulating properties. Subsequently, a 200 nm-thick AlSiCu layer was sputtered on top of the oxide layer with a power of 2250 W (using Timaris FTM). To prevent the formation of the oxide layer, this step was immediately followed by a deposition of 250 nm-thick a-Si:H film by PECVD with 30 sccm of SiH_4_ and 750 sccm of Ar at a temperature of 200 °C. The wafer was diced into smaller samples with dimensions of around 40 mm × 40 mm. The structure undergoes thermal annealing using the Roth and Rau MicroSys 400 equipment in which the heat transfer is performed via shelving. The chamber containing the structure is heated at an average rate of 48.7 °C/min until it reaches a stable temperature of 450 ± 5 °C. These thermal conditions are maintained for a period of 8 h under an average pressure of 2.8·10^−3^ mbar. During the thermal treatment, the silicon atoms diffuse downwards through the metallic layer until the solubility reaches its maximum value. As a result, a continuous poly-Si layer is formed while the metal migrates to the top. The remaining AlSiCu on the surface is removed by a wet-etch process using a standard Al-etch solution (Fujifilm Aluminum Etch 16:1:1:2), allowing for further characterization of the poly-Si film. The composition of the resultant film was inspected by energy-dispersive X-ray spectroscopy (EDX) which confirmed the absence of Al and Cu suggesting that the content of these metals was entirely removed or that its concentration is negligible. The morphology of the crystalline film grown on the Si substrate is presented in the SEM image of Figure 2a, captured using a secondary electron detector (ETD). The picture shows a continuous poly-Si film covered by small particles of residual a-Si, which were not consumed in the crystallization process. The larger particles on the poly-Si film’s surface result from the secondary crystallization [44].

To study the viability of the crystallization on top of polymeric substrates, a Si wafer passivated with 100 nm of SiO_2_ by PECVD was used for mechanical support and was spin-coated with PI HD4110 with a thickness of 7 μm. The SiO_2_ passivation layer can be etched at the end of the process to release the PI substrate from the support wafer, as described in [45]. The bilayer structure of the AlSiCu/a-Si was deposited and annealed under the previously mentioned conditions. The morphology of the resultant poly-Si layer after the metal etch is presented in Figure 2b.

**Figure 3:**
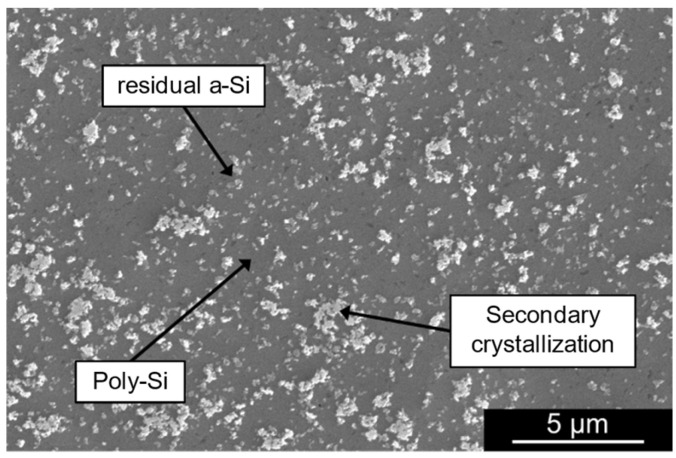
SEM picture of the poly-Si obtained by annealing the AlSiCu (200 nm)/a-Si (250 nm) at 450 °C for 12 h on top of a PI layer.

The image reveals a similar morphology to the film obtained directly on top of the Si wafer, indicating the feasibility of poly-Si growth on top of polymeric substrates. However, the poly-Si islands formed during the secondary crystallization process have larger diameters in the film developed on the Si substrate compared with the film grown on the PI substrate. Additionally, the SEM image shows the presence of voids on the film developed on PI, which suggests that the crystallization process on top of the flexible substrate is slower, as the expansion of the poly-Si islands is in an early stage. To mitigate this void formation in the film, the annealing duration should be extended compared with films grown directly on Si substrates. To validate this hypothesis, an additional test was conducted in which the AlSiCu/a-Si bilayer on PI underwent thermal annealing for 12 h instead of 8 h, with all other parameters unchanged. The SEM image in Figure 3 shows a continuous poly-Si film, confirming that a longer annealing step effectively reduces voids in the grown poly-Si layer. For a fair comparison between poly-Si grown on different substrates, subsequent characterization steps were performed on structures annealed for the same period of 8 h.

### 3.2. Structural Characterization

**Figure 4:**
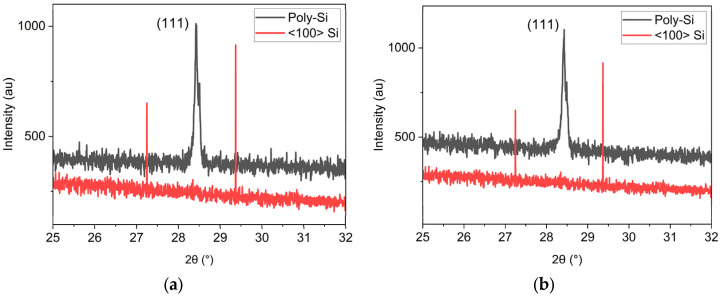
XRD measurements of the poly-Si film obtained showing a crystalline peak in the direction <111> at 2θ = 28.43° (**a**) directly on top of a Si wafer and (**b**) on top of PI HD-4410.

XRD measurements were performed to confirm the crystalline nature of the developed film and estimate its crystallite sizes. The XRD spectrum, shown in Figure 4, provides a comparison between the poly-Si film (black) and the Si wafer (red) used as a substrate, shown in Figure 4a, and as support in the case of poly-Si growth on PI, as in Figure 4b. Both graphs show a peak at 2θ = 28.43°, indicating a preferred crystalline orientation by the (111) plane. The diameter of the crystallites, D, can be calculated using the Scherrer equation (1), where λ is the X-ray wavelength, β is the full width at half maximum (FWHM) of this peak and θ is the Bragg angle.

(1) $$D=\frac{0.9\lambda }{\beta cos(\theta )}$$

With an FWHM of 0.1414°, the crystallites of the poly-Si film developed on the Si substrate have a diameter of approximately 57 nm. As for the film grown on the PI layer, the FWHM of the equivalent peak is 0.1422°, implying that these crystallites have a diameter of around 58 nm.

**Figure 5:**
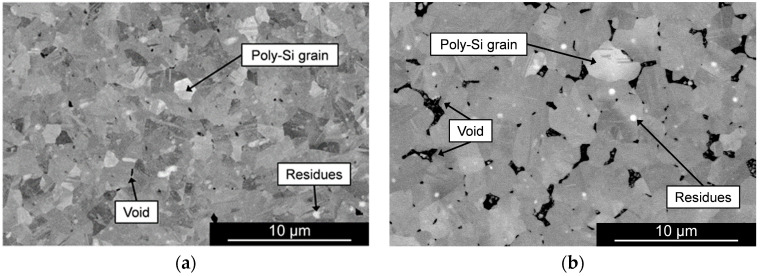
BSE images of the poly-Si film obtained by annealing the AlSiCu (200 nm)/a-Si (250 nm) at 450 °C for 8 h (**a**) on top of a Si-wafer and (**b**) on top of PI 4110-HD layer.

To confirm the polycrystalline structure of the grown silicon film, the samples were inspected by backscattered electron (BSE) detection (using FEI Europe B.V. (Eindhoven, The Netherlands) Quanta 650 FEG). Before the inspection, both samples were cleaned through mechanical polishing from the secondary crystallization islands, exposing the grown poly-Si fully. The BSE images presented in Figure 5 show contrasting regions arising from their distinct crystalline orientation, confirming the polycrystalline nature of the developed film. The residues visible on top of the film are small particles derived from the mechanical polishing process. As shown in Figure 5a, the poly-Si layer grown on the silicon wafer exhibits a dense structure with grain sizes ranging from 1 to 3 μm. In contrast, the poly-Si layer formed on the polyimide substrate, illustrated in Figure 5b, demonstrates a looser arrangement characterized by larger voids among grains, which vary in size from 3 to 7 µm. Larger grains enhance the thin film’s conductivity by reducing grain boundaries per area. However, the presence of voids counterbalances this effect, creating a more resistive film. The difference in grain size between both samples indicates that the nucleation rate of poly-Si on PI is slower than its growth on a silicon substrate. A rapid nucleation rate leads to a higher density of grains, which restricts their lateral growth, resulting in smaller grain sizes.

### 3.3. Design and Microfabrication of Piezoresistor Test Structures

**Figure 6:**
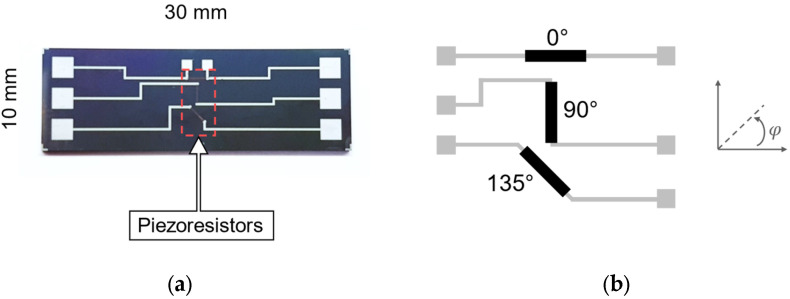
(**a**) Test structure containing three piezoresistors and the conductive paths for external measurements. (**b**) Illustration of the disposition of the three piezoresistors in each device: the top piezoresistor has a rotation of 0°, middle piezoresistor has a rotation of 90° and the bottom piezoresistor has a rotation of 135°.

To evaluate the performance of the poly-Si film for sensor applications, a 30 mm × 10 mm test structure was designed to extract the TCR value, as well as the longitudinal and transversal GF of the material, as illustrated in Figure 6a. The device comprises three resistors, each oriented at a different angle φ relative to the die length (the direction in which the strain will be applied), as shown by the illustration of Figure 6b. One resistor is aligned at φ = 0° for the longitudinal GF extraction, another at φ = 90° for the transversal GF extraction, and a third at φ = 135°, whose behavior is expected to be a combination of both longitudinal and transversal GF. The polycrystalline nature of the developed film was electrically validated by measuring its sheet resistance (Rs). A mean value of 3.28 kΩ/sq was obtained for a film with a thickness of 200 nm, consistent with the literature that reported an Rs of 3.69 kΩ/sq [14]. Given the unknown piezoresistivity and considering the measured Rs, the width of the resistors was held constant at 100 μm, while the length per width (L/w) ratio of the resistor components ranged between 5, 10, and 20. Higher L/w ratios provide better confinement of the stress orientation. Nonetheless, those with smaller ratios have lower electrical resistance, which can facilitate resistance measurements. The defined dimensions lead to resistor components with a mean nominal resistance R_0_ of 20.81 kΩ (L/w = 5), 57.58 kΩ (L/w = 10), and 120.60 kΩ (L/w = 20).

**Figure 7:**
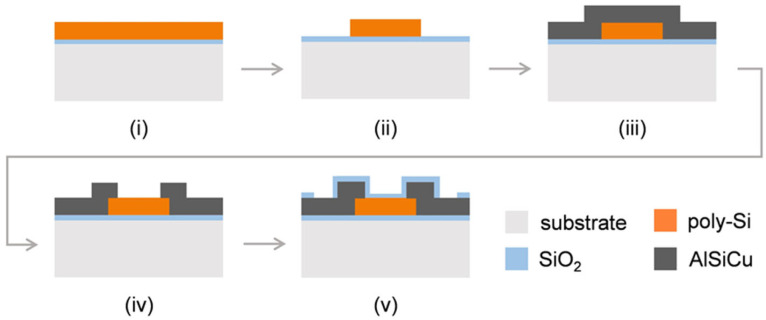
Flow-chart illustration of the fabrication process of the device containing poly-Si piezoresistors for electrical and mechanical characterization of the developed material: (**i**) developed poly-Si; (**ii**) patterning of the poly-Si film; (**iii**) AlSiCu sputtering; (**iv**) AlSiCu patterning and (**v**) deposition and patterning of SiO_2_ passivation layer.

The fabrication of the test structures is illustrated by the flow chart in Figure 7 and consists of the following steps:

(i) The poly-Si thin-film is developed on a Si-wafer following the process described in Section 3.1;
(ii) The polysilicon film is patterned by maskless laser lithography (using Heidelberg Instruments (Heidelberg, Germany) DWL 2000) to define the piezoresistors structure. The unprotected piezoresistive film is etched by Reactive Ion Etching (RIE) (using SPTS (Newport, Wales, UK) Pegasus LPX). The remaining resist is removed by Plasma Asher (using PVA Tepla (Wettenberg, Germany) GIGAbatch 360M);
(iii) A 1 µm thick AlSiCu layer is sputtered to allow the electrical connection between the resistors and the readout electronics;
(iv) The AlSiCu layer is patterned by laser lithography, forming the conductive paths connecting the resistors to the exterior reading circuit, and the exposed AlSiCu is removed by a wet etching solution while the remaining resist was etched by Plasma Asher;
(v) A 100 nm-thick SiO_2_ layer is deposited on top of the structure to act as a protective layer. The oxide is patterned by DWL and etched on the electrical pads area by RIE using SPTS (Newport, Wales, UK) APS, leaving the metal exposed.

## 4. Results and Discussion

### 4.1. Photoconductivity Properties

**Figure 8:**
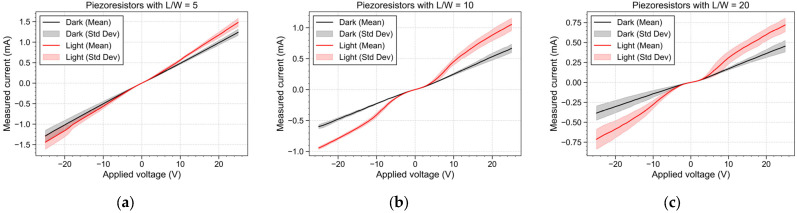
I–V characteristic of the poly-Si piezoresistors with dimensions (**a**) L/W = 5, (**b**) L/W = 10 and (**c**) L/W = 20 extracted under dark and light environments.

The contact between the semiconductor and metallic layers forms a Schottky barrier heterojunction. The fabricated piezoresistors are connected to an aluminum trace on each side, resulting in a structure composed of two Schottky barriers connected by a semiconductor path formed by the piezoresistor between them. This configuration is the foundation of a metal–semiconductor–metal photodetector [46]. Photons are absorbed when a light source is focused on the device, forming an electron–hole pair. Upon applying an electric field to the device, the electron–hole pairs are transported, resulting in a photocurrent that is superimposed to the current that originated from the moving electrons due to the electric field. This additional current decreases the measured resistance, which is proportional to the photon flux that reaches the material. This effect was experimentally validated by applying voltage in a range of −25 V to 25 V and measuring the resultant current both under light and dark environments. The results presented in Figure 8 show that, under dark conditions, the I–V response is approximately linear, typical of a resistive element. However, with incident light, the I–V characteristic becomes non-linear, a response more accentuated for piezoresistors with L/W = 10 and L/W = 20. At low bias voltage (V < 2 V), the generated current is dominated by the dark current while above that threshold the electric potential is sufficient to efficiently transport the photogenerated carriers, leading to a superposition of the dark current and the photocurrent. This behavior highlights the influence of the incident light on the poly-Si conductivity, validating its potential for use in optoelectronic sensing applications.

### 4.2. Long-Term Stability

**Figure 9:**
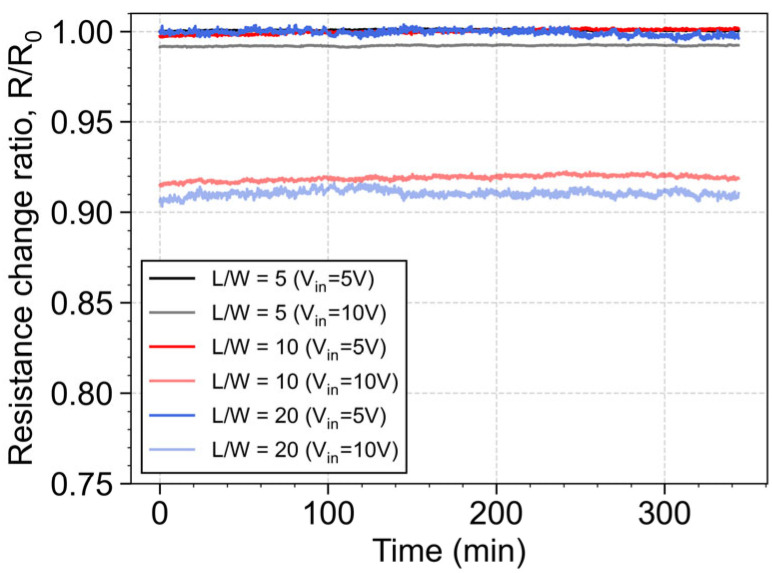
Electrical resistance stability measured for 6 h in a climate chamber at a temperature of 25 °C and humidity of 50% with input voltage signal of 5 V and 10 V for piezoresistors with L/W = 5, 10 and 20 normalized with the nominal resistance at 5 V for each piezoresistor.

To study the long-term stability of the piezoresistors, an input voltage, V_in_, of 5 V and 10 V was applied to the resistive elements, while the current was measured using Keithley Instruments 2410 for a period of 6 h. The tests were performed in a climate chamber (Weiss (Reiskirchen-Lindenstruth/Germany) WKL 34/70) with a constant target temperature of 25 °C and humidity of 50% and by encapsulating the devices in a black box to mitigate external interference during the experimental characterization. The resistance of each piezoresistor is determined by Ohm’s Law and the experimental results are presented Figure 9. The results show that, by increasing the input voltage from 5 V to 10 V, the nominal resistance value decreases proportionally to its initial value, a behavior attributed to self-heating effects. Furthermore, the resistance value of each piezoresistor remains stable over the measurement period, indicating the consistent electrical response over time. It is worth noting that the observed high-frequency variations are primarily due to the precision limitations of the instrumentation equipment used in the characterization as well as thermal fluctuations within the chamber control.

These findings indicate that the developed poly-Si film is reliable and consistent over time under controlled conditions, making the growth of poly-Si by MIC using AlSiCu a viable approach to develop flexible pressure (if temperature is compensated via Wheatstone bridge) and temperature sensors, provided that the material is shielded from mechanical stress.

### 4.3. Temperature Effect

**Figure 10:**
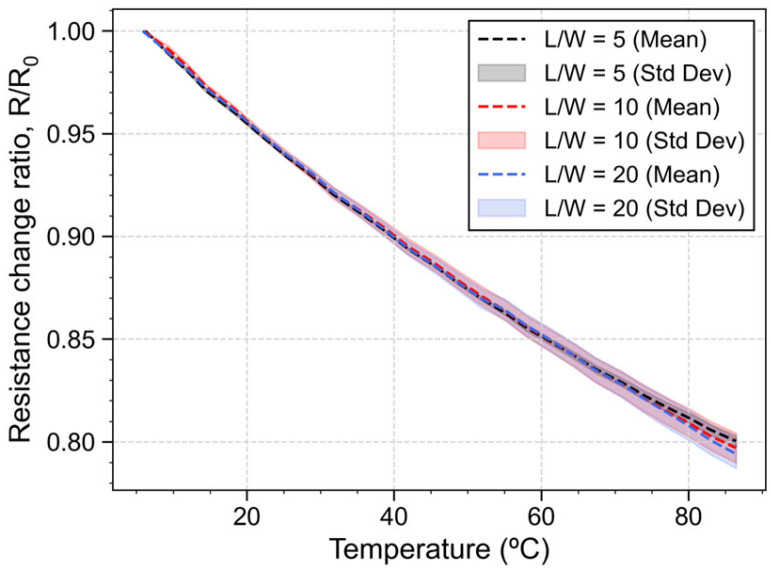
Resistance variation with temperature for piezoresistors with L/W = 5, 10 and 20.

The temperature coefficient of resistance quantifies the influence of environmental temperature on the behavior of piezoresistive material. To extract this coefficient, experimental tests were conducted on nine samples (three samples per L/W ratio) in the climate chamber, where the temperature ranged from 5 °C to 85 °C and had a heating rate of 2.5 °C/min, while the electrical current was acquired for an applied voltage of 1 V to extract the resistance of each piezoresistor using Ohm’s Law. Figure 10 shows that, as the temperature increases, the resistance of the piezoresistors decrease, indicating a mean TCR of −2471 ppm/°C with a non-linearity of 3.37%, calculated as a percentage of the full-range scale (%FS). This outcome shows that the resistors are sensitive to the environment’s temperature, suggesting their potential application in temperature-sensing devices. For other sensing applications, differential configurations, such as the Wheatstone bridge, may be used to mitigate this impact of temperature fluctuations.

### 4.4. Piezoresistive Behavior

**Figure 11:**
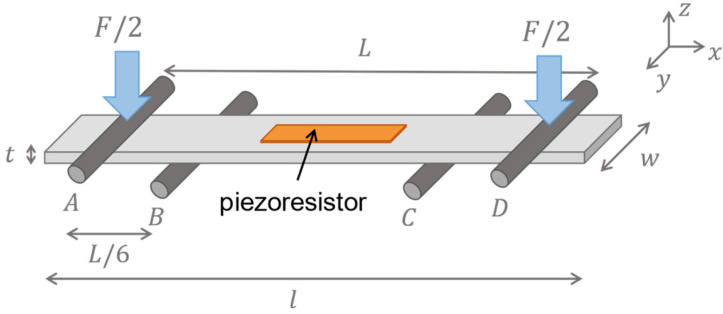
Illustration of a four-point bending set-up for the piezoresistive behavior characterization.

**Figure 12:**
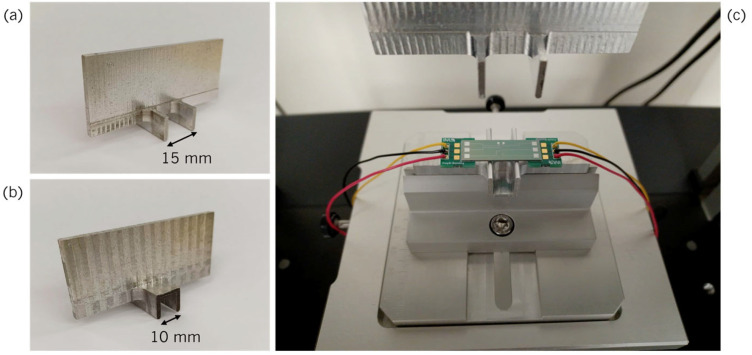
Machined pieces for the (**a**) load beams and (**b**) support beams. (**c**) Positioning of the device in the set up.

As depicted in Figure 11, a four-point bending fixture is employed to apply unidirectional strain to the piezoresistors and to extract their piezoresistive behavior. The proposed bending set-up follows the ASTM E855 08 protocol [47], in which four similar cylindrical structures are symmetrically positioned regarding the center of the chip. Two beams act as support beams upon which the chip is placed, while the other two, known as load beams, are subjected to an external force that is converted to strain by the bending deformation. Following this protocol, the distance between the internal beams is 2/3 of the distance between the external beams, L. In this fixture, the strain applied between the support beams is given by Equation (2).

(2) $$\varepsilon =\frac{FL}{2Ewt^{2}}$$

where, *F* is the applied force, *L* is the distance between the external beams, *E* is the substrate’s Young’s Modulus, *w* is the width, and *t* is the device’s thickness. The tests were conducted on Shimadzu (Kyoto, Japan) AGX-V 10 kN, where forces ranging from 0 to 50 N were applied to study the material’s suitability for a wide range of forces while ensuring that the deformations lay on the elastic regime of the materials composing the test structure. This range of forces translates to a range of strain between 0 and 8 × 10^−4^ according to (2). The structures corresponding to the load and support beams were machined in aluminum. The load beam structure is fixed to the load cell of the instrument, while the support beam structure is fixed to the stage. The devices are positioned on top of the support beams, as shown in Figure 12. The resistance of the piezoresistors is measured using Agilent (Santa Clara, CA, USA) 34410A Multimeter while the force exerted by the load cell induces a bending deformation on the chip. The resistance change that the material undergoes with applied strain is quantified by the *GF* given by Equation (3).

(3) $$GF=\frac{\Delta R/R_{0}}{\Delta l/l_{0}}=\frac{\Delta R/R_{0}}{\varepsilon }\;$$

**Figure 13:**
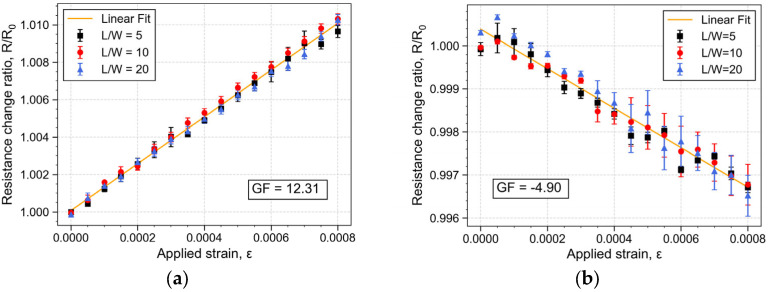
(**a**) Resistance change with strain of the piezoresistors oriented at 0° to extract the longitudinal GF. (**b**) Resistance change with strain of the piezoresistors oriented at 90° to extract the transversal GF.

Figure 13 depicts this resistance change ratio with strain measured for different devices. Figure 13a shows that the longitudinal *GF* has a mean value of 12.31 with a standard deviation, σs, of 0.33 while Figure 13b shows that the transversal *GF* has a mean value of −4.90 with σs = 0.35. These results indicate that piezoresistors oriented toward the applied strain are more sensitive to this external input, which aligns with the higher longitudinal piezoresistive coefficient of Si compared with the transversal coefficient [11]. Additionally, the non-linearity of the piezoresistors response was calculated and it was determined that the longitudinal GF has a non-linearity of 6.35%FS, 2.94%FS and 8.89%FS for piezoresistors with dimensions L/W of 5, 10 and 20, respectively. As for the transversal GF, the non-linearity rises to 9.43%FS, 16.12%FS and 22.90%FS. The measurement of the longitudinal GF comprises strain applied along the length of the piezoresistors, resulting in a more uniform strain distribution and, consequently, in a more predictable response. In contrast, in transversal GF measurements, the strain is applied perpendicularly to the length and aligned to the width. Over a longer length, the resistors are more prone to localized deformations, which can have a cumulative effect as the resistance change depends on the integrated effect of strain along the entire length of the resistor.

Table 1 summarizes the structural and electrical characteristics of poly-Si obtained by the aluminum crystallization method (AIC) as reported in the literature. The longitudinal GF of 12.31 obtained in this study is consistent with previous reports, in which the GF ranges between 5 [29] and 41 [26]. This study also reports a negative GF associated with the sensitivity of the piezoresistors oriented perpendicularly to the direction of applied strain. The negative transversal GF is attributed to the fact that, for p-type Si, the transverse piezoresistive coefficient is negative (=−1.1). In contrast, the longitudinal piezoresistive coefficient is positive (=6.6) according to Y. Kanda [11]. This result sustains the assertion that the Al content in AlSiCu acts as a p+ dopant.

## 5. Conclusions

A poly-Si film was produced on top of a Si wafer and a polymeric substrate by the metal-induced crystallization mediated by AlSiCu at a temperature of 450 °C for 8 h. XRD measurements conducted on both types of samples revealed that the crystallite size of the resultant poly-Si is approximately 57 and 58 nm, respectively, regardless of the substrate used. SEM inspection using the backscattered electron detector confirmed the polycrystalline structure of the developed film. This analysis indicated that the crystallization process is slower when a polymeric substrate is employed, resulting in more dispersed grains with larger sizes (3–7 μm) than the film developed directly on a Si substrate, where the grains have diameters between 1 and 3 μm. Increasing the annealing time of the structure on a PI substrate from 8 h to 12 h effectively reduced voids and consequently improved the quality of the grown poly-Si film. These results demonstrate the potential of this low-temperature MIC process to produce poly-Si films on top of polymeric substrates. It is worth noting that the process developed was performed on industry standard substrates (200 mm Si wafers) and tools, validating the compatibility with large-scale production. Nonetheless, the poly-Si structural and electromechanical properties are highly reliant on the annealing conditions and substrate used, making automation and reproducibility critical for scalability. Additionally, introducing a post-annealing step in the fabrication process could help mitigate potential process-related defects and improve the device’s performance and the overall repeatability.

Test devices containing piezoresistors were fabricated in order to evaluate the polycrystalline film’s thermal stability and piezoresistive response. The piezoresistors and the electrical contacts form a metal–semiconductor–metal junction that is sensitive to light. This effect was quantified by extracting the I–V curve of the different piezoresistors in both dark and light environments. The results obtained with incident light show that for an applied voltage above 2 V, the electric current measured in the piezoresistors is a superposition of the dark current and the photocurrent generated by the transport of the photogenerated carriers. This suggests that the developed film could be further explored in a new range of photonic applications. However, further investigation into the material’s optoelectronic behavior is required. The developed poly-Si exhibited TCR of −2471 ppm/°C, indicating sensitivity to environmental temperature changes. The piezoresistive response was evaluated through a four-point bending setup to apply unidirectional strain to the devices. The experimental results were found to reveal a longitudinal GF of 12.31 and a transversal GF of −4.90. The positive longitudinal GF and the negative transversal GF corroborate that the AlSiCu alloy used in the crystallization process acts as a p+ dopant. Notably, the material properties of the developed poly-Si film are in accordance with those reported in the literature, indicating similar crystalline structure and electrical properties. These findings demonstrate that the metal-induced crystallization process mediated by AlSiCu is a feasible method to produce poly-Si films, achieving a GF at the same level (or potentially higher) as other single-metal crystallization methods. The possibility of using such an alloy as a metal catalyst at low temperatures expands the compatibility of these materials with a wider range of fabrication processes used to create devices like pressure, force, or temperature sensors on rigid and flexible substrates. It is worth noting that, when aiming to develop a piezoresistive sensor, the design should carefully address factors such as sensor geometry and piezoresistor design and location to optimize the sensor performance, ensuring that it effectively leverages the material’s sensitivity. Additionally, because the poly-Si has a significant TCR, the environmental temperature can induce a resistance variation introducing errors in strain sensing applications such as pressure and force sensors. Therefore, this temperature dependence should be considered, especially for environments with significant temperature fluctuations. Self-compensation techniques such as Wheatstone bridge and differential configurations can be implemented to mitigate the temperature effect. On the other hand, to take advantage of this temperature sensitivity in order to develop temperature sensors, the resistance change with strain can be minimized by packaging the sensing elements using stress decoupling encapsulation techniques (soft glue attachment) [48].

In future research, the developed poly-Si will be explored in flexible piezoresistive sensors. Nonetheless, this requires adjustments to the test structure design to accommodate high mechanical deformations, as well as the development of a dedicated characterization setup.

## Figures and Tables

**Table 1:** Summary of reported poly-Si structural and electrical characteristics obtained by the AIC method.

Reference	Annealing Conditions	Poly-Si Thickness (nm)	Sheet Resistance (kΩ/sq)	Crystallites Size (nm)	GF	TCR (ppm/°C)
[30]	400 °C for 1 h	200	2.5	49	10.316	−2700
[14]	400 °C for 1 h	200	3.69	21.6	-	−2500 (PI substrate) −2800 (glass substrate)
[29]	550 °C for 6 h	1000	4	30–50	5 (transversal) 18 (longitudinal)	-
[31]	400 °C for 1.5 h	500	1.4–2	30–47	6.6–11.7	-
[26]	300 °C for 1 h	150–200	-	22–25	41 ± 3	-
Present work	450 °C for 8 h	200	3.28	≈58	−4.90 (transversal) 12.31 (longitudinal)	−2471

## Data Availability

The original contributions presented in this study are included in the article. Further inquiries can be directed to the corresponding authors.
